# Inappropriate ICD Shocks - When Monitoring Zones Do More Than Monitor

**DOI:** 10.1016/s0972-6292(16)30673-8

**Published:** 2013-09-01

**Authors:** Reinder Evertz, Emilce Trucco, Jose Maria Tolosana, Elena Arbelo

**Affiliations:** Arrhythmia Section, Cardiology Department. Thorax Institute, Hospital Clinic. Barcelona, Catalunya, Spain.

**Keywords:** Inappropriate ICD Shocks, Monitoring Zones

## Abstract

The ventricular tachycardia (VT) monitoring zone in implantable cardioverter defibrillators (ICDs) is usually programmed to detect slow VTs. However, it is not well known whether programming this zone can affect the ICD arrhythmia redetection or confirmation criteria. We report two cases of inappropriate ICD shocks due to the programming of a slow VT monitoring zone in the same device model.

The ventricular tachycardia (VT) monitoring zone in implantable cardioverter defibrillators (ICDs) is usually programmed to detect slow VTs. However, it is not well known whether programming this zone can affect the ICD arrhythmia redetection or confirmation criteria. We report two cases of inappropriate ICD shocks due to the programming of a slow VT monitoring zone in the same device model.

## Case 1

A 62-year-old man presented at the outpatient clinic for device control after receiving an ICD shock. A single-chamber ICD (Current VR, St Jude Medical) had been implanted two years earlier for primary prevention while the patient was having symptoms of NYHA class III heart failure due to a dilated cardiomyopathy with left ventricular ejection fraction of 23%. Three tachycardia zones were programmed: ventricular fibrillation (VF) at a heart rate (HR) ≥200 bpm (300 ms), VT (VT-2) at HR between 171 and 200 bpm (350-300 ms), and a slow VT zone (VT-1) as a monitoring zone between 120 and 171 bpm (500-350 ms). Tachycardia was diagnosed once 12 recorded intervals were within any of the tachycardia zones. Supraventricular tachycardia (SVT) discriminators were programmed "ON" in the monitor zone using the nominal settings: morphology criterion (5 of 8 complexes have ≥ 60% morphology match with the sinus complex), sudden onset (programmed to 100 ms), and stability criterion (variability < 80 ms).

**Figure 1:**
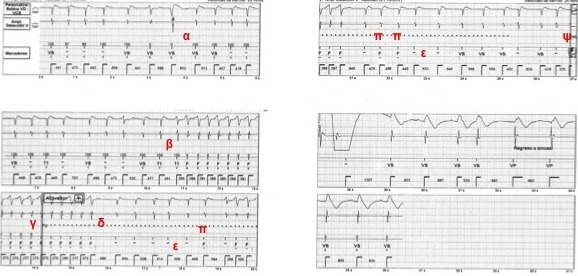
ICD window of patient 1. α Atrial fibrillation. β Onset of a non sustained tachycardia (probably VT) in the VF zone. γ VF episode declared with the start of capacitors charging. δ Tachycardia terminated spontaneously. ε Intervals not classified (-) because both "binning" criteria were not met (the interval < 500 ms (monitor zone) or the average of the 3 preceding intervals falling within the tachycardia zone). Intervals classified as VF (F) because of meeting both "binning" criteria (< 500 ms and the average) of which π are misclassified. Only four intervals met both "binning" criteria for SR (VS) (> 500 ms and the average of the 3 preceding intervals falling outside the tachycardia zone), so therapy could not be diverted and ψ an inappropriate, synchronized shock was delivered.

The patient's baseline rhythm was atrial fibrillation (AF). A VF episode was diagnosed once 12 consecutive intervals were detected in the VF zone (interval of 270-289 ms), and capacitors charging began. Despite spontaneous termination of tachycardia during charging, a shock of 36J was delivered. Upon interrogation both device and lead malfunction were ruled out. The tracing of the device interrogation of the episode is depicted in Figure 1.

During charging of the capacitors, the device is programmed by default to terminate therapy after 5 intervals of sinus rhythm (SR), meaning 5 intervals below the programmed tachycardia zones (in this specific case below the monitor zone of HR 120-171 bpm). The device does this through the process of "binning": an interval is classified on the basis of its duration (in this patient, a cut-off value of 500 ms) and the average of the 3 preceding ones.

As can be seen in the figure, the first 5 intervals during charging are properly classified as VF (marked as F). Thereafter the tachycardia terminates and AF continues. The irregular and rather fast ventricular response with intervals in the monitor zone explain why only 4 beats could be binned as SR. Three intervals were probably misclassified as VF (marked as ∞) because they met both binning criteria, but the morphology was unchanged (a criterion the device does not use in this zone). Intervals >500 ms can be detected, but were not classified (marked as -) because they did not meet the second criterion for binning (the average of the preceding beats). In the absence of 5 intervals that could be classified as SR, at the end of charging a synchronized shock was delivered.

## Case 2

**Figure 2:**
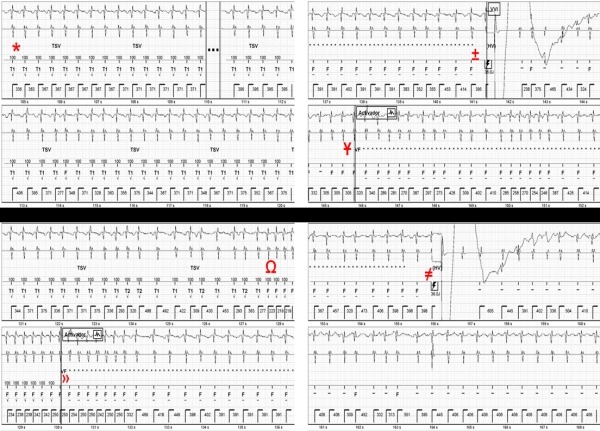
ICD window of patient 2 with the first two of six inappropriate shocks. * Episode correctly classified as SVT. Ω Sudden increase of the heart rate, reaching the VF zone. » VF episode declared with the start of capacitors charging. ± First inappropriate shock with a heart rate between 132 and 155 bpm (no 5 beats could be classified as SR). Ұ Inappropriate post shock redetection of VF due to the ongoing tachycardia and 6 beats classified as VF. ≠  Second inappropriate shock. After the second shock the heart rate slowed down, but remained in the VT-1 zone, prolonging the redetection period as long as no 5 beats can be classified as SR.

A 40-year-old woman with arrhythmogenic right ventricular dysplasia received a single-chamber ICD (Current VR, St Jude Medical) for secondary prevention. Three tachycardia zones were programmed: VF at HR ≥214 bpm (<280 ms), VT-2 with HR between 184 and 214 bpm (326-280 ms), and a VT-1 as a monitoring zone between 120 and 183 bpm (500-326 ms). Nominal SVT discriminators were programmed "ON" and SVT criteria timeout and VT therapy timeout were programmed "OFF". The patient presented at the outpatient clinic after receiving 6 ICD shocks. Device and lead dysfunction were ruled out. The tracing of the device interrogation of the episode is depicted in Figure 2.

The episode started with a SVT at 155 bpm, which was appropriately discriminated for 127 seconds. However, HR suddenly increased, reaching the VF zone. After the detection of 12 VF intervals, a VF episode was declared. As in the first case, therapy would have been withheld if during charging 5 SR beats had been detected (R-R interval >500 ms). During charging, the HR slowed, reaching the VT-1 zone. Because the HR remained in the tachycardia zone no intervals could be classified as SR and the first inappropriate ICD shock was delivered. After the ICD shock, the nominal post-shock redetection criteria require 5 beats in SR (<120 bpm or >500 ms) to finalize the episode; 6 intervals classified as VF (marked as F) are needed to redetect the tachycardia as VF (in this case programmed to be in the VT-2 or VF zone, again meeting both binning criteria). The intervals within the monitor zone can neither be classified as SR nor as VF (marked as -). After the first shock a fast redetection of VF occurred because 6 of the 11 intervals were inside the VT-2 or VF zone. The capacitors were charged again and a second inappropriate shock was delivered. After the second shock the HR slowed but remained above 120 bpm (VT-1), thus prolonging the redetection period until the relevant criteria were satisfied. Unfortunately, due to the ongoing tachycardia, the episode was not ended and every time 6 VF intervals were classified, therapy was delivered. This patient received 6 consecutive shocks, until therapy was exhausted. The total duration of the episode was 355 seconds.

## Discussion

Both case reports of patients with different characteristics show inappropriate ICD shocks with a St Jude Medical Current VR ICD due to the specific arrhythmia detection and redetection programming, as described in other St Jude ICD models [1].

The programming of a slow VT (monitoring) zone in combination with the use of binning prevented the diversion of therapy in these patients. The first patient had an inappropriate shock due to therapy not being diverted during charging of the capacitor because only 4 intervals were binned as SR, as the patient was in AF with R-R intervals inside the monitoring zone (<500 ms). The second patient had inappropriate shocks because the redetection time was prolonged during an ongoing SVT in the monitoring zone and, again, no 5 intervals could be classified as SR. Redetection of 6 nonconsecutive intervals in the VT-2 or VF zone resulted in ICD shocks.

To prevent these inappropriate shocks from occurring in this particular ICD model, it is reasonable to avoid programming a VT monitoring zone and to consider a higher VF (and VT-2) threshold to minimize the risk of binning a fast interval. Programming sinus redetection at 3 instead of 5 bins increases the likelihood of earlier closure of the confirmation period during charging as well as during the post-shock redetection period. This adjustment would have successfully diverted therapy in the first case. On the other hand, increasing the number of intervals required to detect tachycardia as VF could have prevented the declaration of an episode and initiation of capacitor charging in both cases. These changes in the tachycardia detection and redetection programming may decrease the possibility of an inappropriate shock, with a slightly elevated risk of undertreatment [2].

In its newer family of devices, St Jude Medical changed the programming of the monitor zone. During charging and during redetection, intervals in this zone will not be classified as FV, as the monitor zone is no longer a true tachycardia zone. During the post-shock redetection period intervals in the monitor zone will be classified as SR and after 5 intervals noted as such, the episode will be terminated. When a tachycardia in the monitor zone continues this will open a new episode, noted as monitor zone. These cases underline the importance of setting adequate parameters on the VT monitoring zone to avoid serious consequences for patients with implantable devices [3].
